# Supplementary material for: Splice-Junction-Based Mapping of Alternative Isoforms in the Human Proteome
Source: Cell Rep. Author manuscript; Available in PMC 2020 Jan 15. (PMC6961840; doi:10.1016/j.celrep.2019.11.026)
Supplement: 2 [file NIHMS1546469-supplement-2.zip › DF1/PXD010154/Prostate/Prostate_19_SYPL1_ETSLHSPSNTSAPHSQGGIPPPTGL.pdf]

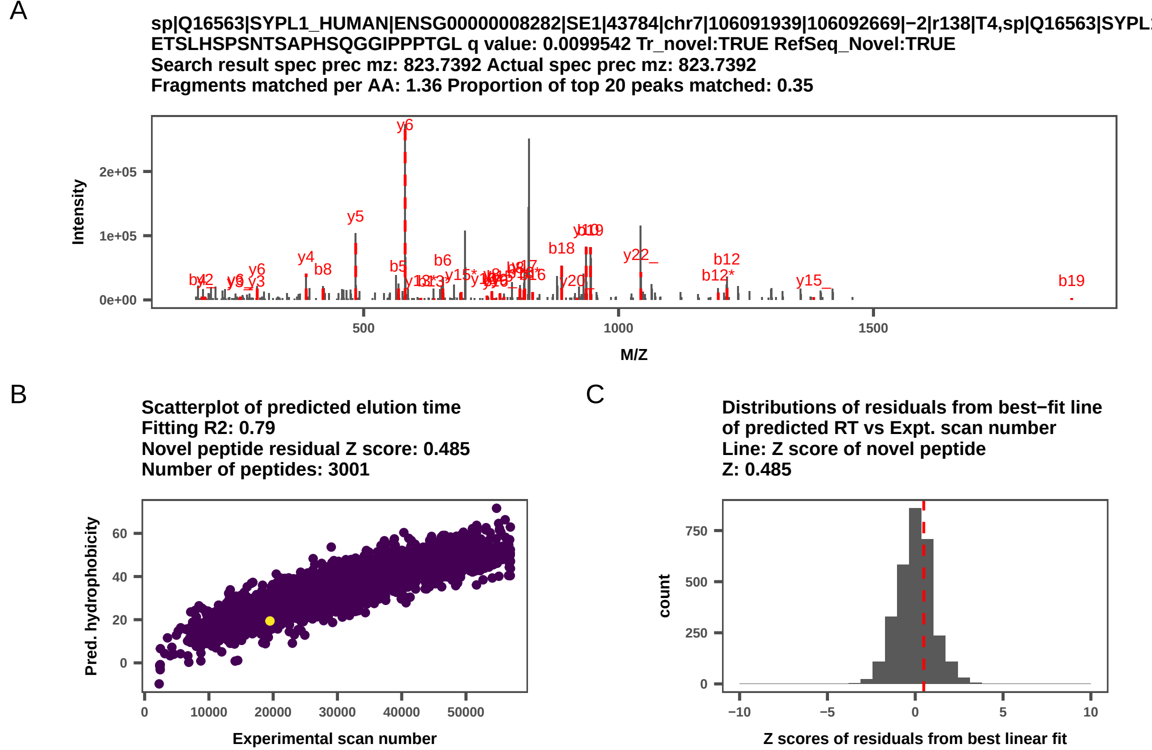
A
sp|Q16563|SYPL1_HUMAN|ENSG00000008282|SE1|43784|chr7|106091939|106092669|−2|r138|T4,sp|Q16563|SYPL
ETSLHSPSNTSAPHSQGGIPPPTGL q value: 0.0099542 Tr_novel:TRUE RefSeq_Novel:TRUE
Search result spec prec mz: 823.7392 Actual spec prec mz: 823.7392
Fragments matched per AA: 1.36 Proportion of top 20 peaks matched: 0.35
Intensity
2e+05
1e+05
0e+00
y6
y5
y4
b8
b5
b6
y15*
b18
y20
y22_
b12
b12*
y15_
b19
500
1000
1500
M/Z
B
Scatterplot of predicted elution time
Fitting R2: 0.79
Novel peptide residual Z score: 0.485
Number of peptides: 3001
Pred. hydrophobicity
60
40
20
0
0
10000
20000
30000
40000
50000
Experimental scan number
C
Distributions of residuals from best−fit line
of predicted RT vs Expt. scan number
Line: Z score of novel peptide
Z: 0.485
count
750
500
250
0
−10
−5
0
5
10
Z scores of residuals from best linear fit
